# IL-17RC is critically required to maintain baseline A20 production to repress JNK isoform-dependent tumor-specific proliferation

**DOI:** 10.18632/oncotarget.17820

**Published:** 2017-05-11

**Authors:** Chi Yan, Yang Lei, Tong-Jun Lin, David W. Hoskin, Averil Ma, Jun Wang

**Affiliations:** ^1^ Canadian Center for Vaccinology, Halifax, Nova Scotia, Canada; ^2^ Department of Microbiology and Immunology, Faculty of Medicine, Dalhousie University, Halifax, Nova Scotia, Canada; ^3^ Department of Pediatrics, Faculty of Medicine, Dalhousie University, Halifax, Nova Scotia, Canada; ^4^ Department of Pathology, Dalhousie University, Halifax, Nova Scotia, Canada; ^5^ IWK Health Centre, Halifax, Nova Scotia, Canada; ^6^ Department of Medicine, University of California, San Francisco, California, USA

**Keywords:** IL-17 receptor C, A20, JNK1, JNK2, B16 melanoma

## Abstract

The IL-17/IL-17R axis has controversial roles in cancer, which may be explained by tumor-specific results. Here, we describe a novel molecular mechanism underlying IL-17RC-controlled tumor-specific proliferation. Triggered by IL-17RC knockdown (KD), B16 melanoma and 4T1 carcinoma cells inversely altered homeostatic tumor proliferation and tumor growth *in vitro* and *in vivo*. In contrast to the existing dogma that IL-17RC-dependent signaling activates the JNK pathway, IL-17RC KD in both tumor cell lines caused aberrant expression and activation of different JNK isoforms along with markedly diminished levels of the ubiquitin-editing enzyme A20. We demonstrated that differential up-regulation of JNK1 and JNK2 in the two tumor cell lines was responsible for the reciprocal regulation of c-Jun activity and tumor-specific proliferation. Furthermore, we showed that A20 reconstitution of IL-17RCKD clones with expression of full-length A20, but not a truncation-mutant, reversed aberrant JNK1/JNK2 activities and tumor-specific proliferation. Collectively, our study reveals a critical role of IL-17RC in maintaining baseline A20 production and a novel role of the IL-17RC-A20 axis in controlling JNK isoform-dependent tumor-specific homeostatic proliferation.

## INTRODUCTION

IL-17A is a pleiotrophic inflammatory cytokine that has multi-faceted roles in cancer [1, 2]. A wide range of cell types, including type 17 CD4^+^ T helper cells (Th17), γδ T cells, and innate lymphoid cells [3–5], as well as tissue structure cells like Paneth cells of the intestinal epithelium [6], are capable of producing IL-17A. Clinical studies have detected IL-17A-producing cells in a variety of human cancer samples and increased IL-17A level is reported to associate with poor prognosis in some studies, but improved prognosis in other reports [7]. In dealing with the controversy, the role of IL-17A in tumorigenesis has been postulated to depend on multiple factors including the specific tumor type and the cellular sources of IL-17A [2, 7]. Various molecular and cellular mechanisms mediate the pro-tumor and/or anti-tumor functions of IL-17A. Specifically, IL-17A can indirectly control tumorigenesis via regulating inflammatory responses, angiogenesis and anti-tumor immunity [2, 8–10], and directly influence tumor growth and neoplastic transformation [11–14]. Notably, while IL-17A is reported to induce neoplastic transformation and proliferation of some tumor cell lines *in vitro* [11, 12], it fails to do so in many other tumor cell lines [12, 14]. This phenomenon suggests that the IL-17A signaling in tumor cell proliferation is tightly regulated; however, the molecular mechanisms underlying the tumor-specific proliferation induced by IL-17A are largely understudied.

A receptor complex containing IL-17RA and RC subunits is essential for IL-17A signaling [15–17]. The binding of IL-17A, or its close family member IL-17F, to the IL-17RA-RC complex recruits the intracellular adaptor protein Act1, which activates TRAF6 leading to activation of nuclear factor kappa B (NF-κB) [15–17] and selective activation of mitogen-activated protein kinase (MAPK) pathways, particularly c-Jun NH2-terminal kinase (JNK) pathway, in different target cells [18–21]. IL-17A also induces the phosphatidylinositide 3-kinases and protein kinase B (PI3K/Akt) pathway in epithelial cells and fibroblasts [22, 23]. Consequently, IL-17A induces synthesis of various gene products, including pro-inflammatory cytokines, chemokines, matrix metalloproteinases and growth factors, to mediate diverse biological functions in autoimmunity, inflammation, host defense, and cancer [15, 16]. Although IL-17RA and IL-17RC subunits operate in concert to mediate IL-17A signaling, IL-17RC possesses unique intracellular domains that are involved in modulating IL-17A-induced signaling [24]. Given that IL-17RA and IL-17RC are differentially expressed by hematopoietic and non-hematopoietic cells [15], the ratio of IL-17RA/IL-17RC is postulated to control IL-17A-induced cytokine response in a cell-type-dependent manner [15]. However, the mechanism(s) by which IL-17RC may regulate cell-type-dependent proliferation remains elusive.

In the past decade, multiple signaling molecules have been demonstrated to negatively or positively regulate IL-17A-induced responses [17]. A key negative inhibitor of IL-17A-induced signaling is the ubiquitin-editing enzyme A20 [25]. A20, encoded by the gene TNFα-induced protein 3 (*TNFAIP3*), is a potent negative regulator of the NF-κB pathway and has a vital role in controlling inflammation and apoptosis [26, 27]. Although initially described as a negative feedback inhibitor of TNFα-induced signaling, A20 also inhibits TLR, IL-1R, and Nod-like receptor pathways in addition to the IL-17R pathway [25–27]. A20-deficient mice exhibit multi-organ inflammation and perinatal lethality due to uncontrolled NF-κB activity [28, 29]. Biochemically, A20 is an ubiquitin-editing enzyme that exhibits de-ubiquitination, E3 ligase, and ubiquitin-binding activities. The N-terminal ovarian tumor domain of A20 is responsible for its de-ubiquitinating activity, whereas the seven zinc fingers at the C-terminus mediate E3 ubiquitin ligase and ubiquitin-binding activities [26]. While activation of NF-κB is controlled by both K48- and K63-polyuniquitination of upstream signaling proteins, A20 turns off NF-κB by modulating both types of ubiquitination [25, 26, 30]. A20 also inhibits IRF-3-dependent gene production [26, 31], TNF-induced apoptosis and IL-17A-induced IL-6 production via inhibition of the JNK pathway [25, 32]. Furthermore, A20 inhibits Wnt signaling and reduced A20 expression is associated with human colorectal cancer development [33, 34]. Given the vital role of A20 in controlling inflammation, it is conceivable that steady-state levels of A20 dictate the overall magnitude of inflammatory signals. While many pro-inflammatory stimuli can induce A20 production during inflammatory responses [26, 27, 31], it is less clear how A20 is maintained under steady-state conditions.

JNKs, also known as stress-activated protein kinases, are activated by a wide range of stimuli, including pro-inflammatory cytokines and stress signals, such as UV-irradiation and starvation [35, 36]. There are three members in the JNK family (JNK1-JNK3), among which JNK1 and JNK2 are expressed broadly and JNK3 is expressed predominantly in the brain, testes, and heart. Upon stimulation, JNKs phosphorylate and activate a number of nuclear and non-nuclear proteins, such as the transcription factor activator protein-1 (AP-1), which is formed by dimerization of the Jun proteins (c-Jun, JunB, JunD) with the Fos proteins (c-Fos, FosB, Fra-1, Fra-2) to control cell proliferation, differentiation, cell death, inflammation, and cell metabolism [35, 36]. Specifically, the transcriptional activity of c-Jun is controlled by phosphorylation of serine-63 and/or serine-73 residues, as well as the level of c-Jun expression [37]. While the two major JNK proteins—JNK1 and JNK2 have overlapping roles in various biological functions such as promoting cytokine production, they have distinct roles in controlling cell proliferation [35, 38, 39]. JNK1 and JNK2 oppositely regulate the stability and activation of c-Jun and control c-Jun-dependent proliferation in fibroblasts, with JNK1 promoting and JNK2 inhibiting cell cycle progression [38]. The epidermis isolated from JNK2^−/−^ and JNK1^−/−^ mice is hyperplastic and hypoplastic, respectively [39]. In addition to controlling cell proliferation, JNK1, but not JNK2, promotes UV-induced apoptosis in skin cancer cells [40]. Conversely, JNK2 constitutively suppresses JNK1-mediated apoptosis in multiple human cancer cell lines and promotes basal tumor cell survival [41, 42]. While the JNK pathway is prevalently viewed as one of the IL-17A-induced signaling pathways, the role of JNK1 and JNK2 isoforms in mediating IL-17/IL-17R-controlled tumor-specific responses has never been investigated.

In this study, we investigate the role of IL-17RC in two different murine cell lines—B16 melanoma and 4T1 mammary carcinoma, using a lentivirus vector-mediated shRNA knockdown (KD) approach. Surprisingly, IL-17RC silencing in two cell lines has divergent impact in cellular proliferation. We demonstrate that IL-17RC is critically required for maintaining basal levels of the ubiquitin-editing enzyme A20, which represses homeostatic proliferation of tumor cells in a JNK1/JNK2 isoform-dependent manner. Our work reveals a complex molecular mechanism underlying the tumor-specific proliferation that is controlled by the IL-17R-A20 axis.

## RESULTS

### IL-17RC silencing alters tumor growth *in vitro* and *in vivo* in a tumor-dependent manner

**Figure 1:**
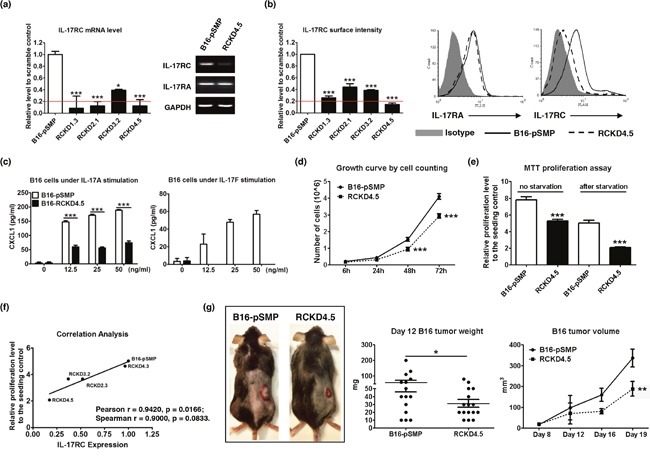
Specific knockdown of IL-17RC expression in B16 melanoma cells attenuates tumor growth *in vitro* and *in vivo* B16 cells were transduced with retroviral vectors containing shRNAs against IL-17RC or random sequences. IL-17RC expression by different knockdown sub-clones was determined by qRT-PCR and RT-PCR **(a)** and flow cytometry **(b)**. The threshold of gene expression for selecting the best knockdown is shown as a red line. **(c)** CXCL1 production upon IL-17A and IL-17F stimulation was determined by ELISA. **(d-f)** Cell growth was measured by direct cell counting and MTT assay with or without serum-free starvation treatment and correlation analysis with IL-17RC expression. **(g)** Tumor weight and volume of B16-IL-17RCKD and B16-pSMP control cells in C57BL/6 mice was determined. All values are presented as the mean ± SEM of 3 independent experiments for *in vitro* studies **(a-f)**, or the mean ± SEM of 5-15 mice per group per time point for *in vivo* studies **(g)**. *p ≤ 0.05; **p ≤ 0.01; ***p ≤ 0.001; statistical analysis was compared with the pSMP control.

To examine the role of IL-17A/IL-17R in controlling cancer cell proliferation, we selected two well-characterized tumor cell lines, B16 melanoma and 4T1 mammary carcinoma, for our study and created IL-17RCKD clones using retroviral shRNA constructs alone with pSMP control vector. Notably, all four shRNA constructs were able to significantly reduce IL-17RC expression at mRNA and protein levels (Figure 1a, 1b). Representative clones that had >80% IL-17RC reduction and marginal change in IL-17RA expression were selected for further characterization. Compared to the pSMP control cells, B16-RCKD clones, as represented by the RCKD4.5 clone, produced significantly less CXCL1 upon IL-17A and IL-17F stimulation (Figure 1c), demonstrating a functional impairment of the IL-17A/F-induced signal transmission in RCKD clones. Of interest, we noticed that B16-RCKD cells grew significantly slower than B16-pSMP control cells, which was measured by cell counting and MTT proliferation assay under normal culture condition and after serum starvation (Figure 1d, 1e). Correlation analysis revealed that cell proliferation was significantly and positively correlated with the level of IL-17RC expression in B16-RCKD clones (Figure 1f). When the tumor cells were subcutaneously inoculated into C57BL/6 mice, the resulting B16-RCKD tumors were significantly smaller by volume and by weight compared to B16-pSMP tumors (Figure 1g). Together, our data suggest a positive role of IL-17RC in supporting the proliferation of B16 melanoma cells *in vitro* and *in vivo*.

**Figure 2:**
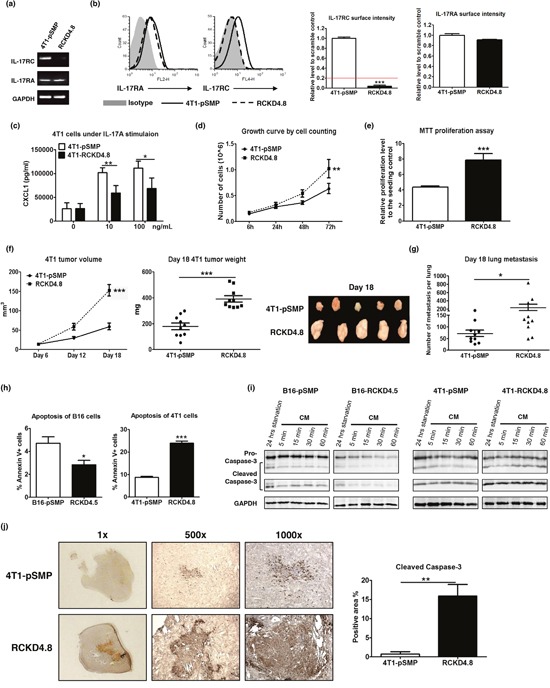
Specific knockdown of IL-17RC expression in 4T1 cells promotes tumor proliferation and tumor invasiveness *in vitro* and *in vivo* despite increased stress-induced apoptosis 4T1 cells were transduced with retroviral vectors containing shRNAs against IL-17RC or random sequences. **(a-b)** IL-17RA and RC expression from a representative IL-17RCKD clone (RCKD4.8) and the pSMP control of 4T1 cells were examined by RT-PCR and flow cytometry. The threshold of gene expression for selecting the knockdown clones is shown as a red line. **(c)** CXCL1 production upon IL-17A stimulation was determined by ELISA. **(d-e)** Cell growth was measured by direct cell counting and MTT assay with serum-free starvation treatment. **(f-g)** Tumor volume, weight and lung metastasis of 4T1-IL-17RCKD and 4T1-pSMP control in Balb/c mice were determined. **(h-i)** RCKD and pSMP control subclones of B16 and 4T1 cells were starved in serum-free medium for 14 hours and recovered in complete medium (CM) for different periods of time. The rates of apoptosis were determined by Annexin V staining 1 hour following CM **(h)**. Whole-cell extracts were harvested and immunoblotted with antibodies to detect pro- and cleaved-caspase-3. GAPDH was used as a loading control **(i)**. **(j)** Representative images and quantitative results of cleaved-caspase-3 protein levels observed from day 18 in 4T1 tumors by immunohistochemistry. All values are presented as the mean ± SEM of 3-5 independent experiments for *in vitro* studies **(a-e, h)**, or the mean ± SEM of 5-10 mice per group per time point for *in vivo* studies **(f-g, j)**. *p ≤ 0.05; **p ≤ 0.01; ***p ≤ 0.001; statistical analysis was compared with the pSMP control.

Representative RCKD clones with profound IL-17RC reduction at mRNA and protein levels were also created in 4T1 cells (Figure 2a, 2b, 2c). Surprisingly, the loss of IL-17RC expression in 4T1 cells directly promoted tumor cell growth in culture. As shown in Figure 2d, 2e, the representative 4T1-RCKD4.8 clone displayed a 1.5- to 2-fold increase in proliferation rate compared to the 4T1-pSMP control *in vitro*. Furthermore, the primary 4T1-RCKD tumors were approximately 2.5-fold larger than 4T1-pSMP cells at day 18 post-tumor inoculation (Figure 2f), and generated significantly more lung metastases (Figure 2g). Therefore, in sharp contrast to its role in B16 melanoma, IL-17RC is a strong negative regulator of 4T1 homeostatic proliferation and invasiveness *in vitro* and *in vivo*.

To investigate whether IL-17RC-controlled tumor growth was associated with altered apoptosis, flow cytometric analyses were conducted to measure the rates of serum starvation-induced apoptosis in RCKD clones and pSMP controls. Notably, RCKD reduced the percent of annexin V-positive B16 cells, but markedly increased the apoptosis of 4T1 cells (Figure 2h). We also measured caspase-3 activity using Western blotting to verify the results (Figure 2i). Consistent with the flow cytometric analyses of annexin V-stained cells, the levels of total and cleaved caspase-3 were reduced in B16-RCKD cells compared to B16-pSMP controls; in sharp contrast, the amounts of cleaved caspase-3 were dramatically increased in 4T1-RCKD cells; however, the total levels of caspase-3 were comparable among 4T1-RCKD cells and 4T1-pSMP control cells. Similar to the *in vitro* observations, the levels of cleaved caspase-3 were significantly increased in 4T1-RCKD tumor sections compared to their pSMP counterparts (Figure 2j). Therefore, our data suggest that IL-17RC has divergent roles in controlling homeostatic proliferation and stress-induced apoptosis in different tumor types. Notably, despite its impact on stress-induced apoptosis, IL-17RC-controlled homeostatic proliferation appears to ultimately dictate the invasiveness of tumor cells *in vitro* and *in vivo*.

### IL-17RC silencing induces acquired-activation of different JNK isoforms in different tumor cells, which differentially regulates c-Jun-dependent homeostatic proliferation

**Figure 3:**
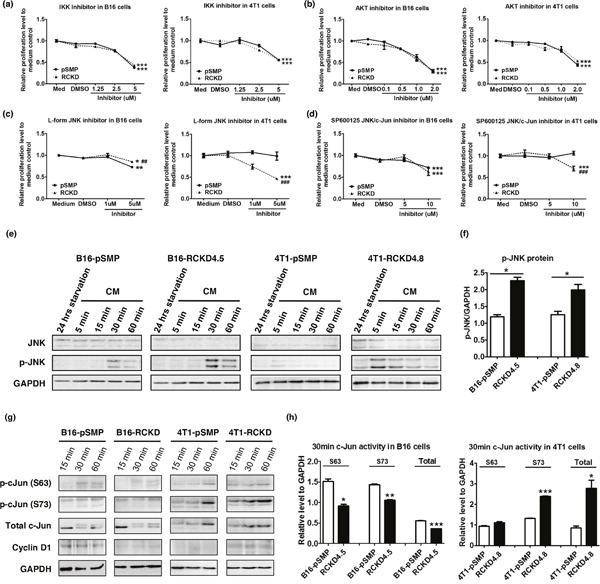
IL-17RC silencing results in acquired-JNK activation but distinct c-Jun activities in B16 and 4T1 cells **(a-d)** IL-17RCKD and pSMP controls of B16 and 4T1 cells were treated with DMSO or one of the inhibitors for 48 hours. Cell proliferation was measured by MTT assay. All values are presented as the mean ± SEM of 4-6 replicates in two independent experiments. *p ≤ 0.05; **p ≤ 0.01; ***p ≤ 0.001; statistical analysis was compared with the DMSO control. ^##^*p* ≤ 0.01; ^###^*p* ≤ 0.001; statistical analysis was compared with the pSMP control. **(e-h)** Whole-cell extracts were harvested and immunoblotted to detect total or phosphorylated proteins as indicated. GAPDH was used as a loading control. Scanning densitometry of relative phospho (p)-JNK protein levels at 60 minutes post CM recovery was performed. All values are presented as the mean ± SEM of 4-6 replicates in two independent experiments; *p ≤ 0.05; **p ≤ 0.01; ***p ≤ 0.001; statistical analysis was compared with the pSMP control.

To identify the specific signaling pathway(s) responsible for altered homeostatic proliferation of RCKD clones in B16 and 4T1 cells, well-characterized pharmacologic inhibitors were used to block NF-κB, PI3K-Akt and MAPK pathways in cell culture. Notably, the homeostatic proliferation of IL-17RCKD clones and pSMP controls of both B16 and 4T1 cells was significantly inhibited by KIN001-102 and BMS-34554, specific inhibitors for Akt and Ikappa B kinase (IKK), respectively (Figure 3a, 3b), suggesting that Akt and NF-κB pathways provide survival signals for both B16 and 4T1 cells under steady-state conditions. However, pSMP control and IL-17RCKD B16 and 4T1 cells exhibited similar sensitivities to the Akt and IKK inhibitors (Figure 3a, 3b), indicating that IL-17RC-controlled cell proliferation is mediated neither by Akt nor NF-κB signals. Of interest, 4T1 and B16 pSMP control cells, as well as their corresponding RCKD clones, displayed distinct sensitivities to the inhibitors targeting the JNK/c-Jun pathway (Figure 3c, 3d) while responding similarly to extracellular signal-regulated kinase (ERK) and p38 MAPK inhibitors (data not shown). Specifically, L-form JNK inhibitor was able to inhibit proliferation of B16-pSMP cells, but not 4T1-pSMP cells; however, IL-17RC silencing resulted in reduced sensitivities to L-form JNK inhibitor in B16-RCKD cells, but markedly enhanced sensitivities in 4T1-RCKD cells (Figure 3c). Furthermore, the 4T1-RCKD clone displayed obvious sensitivities to SP600125 JNK/c-Jun inhibitors, which showed no activity in 4T1-pSMP cells, and comparable activities in B16-pSMP and B16-RCKD clones (Figure 3d), highlighting a role of IL-17RC in suppressing homeostatic JNK/c-Jun activation in 4T1 but not B16 cells. To verify this finding, we examined total JNK/c-Jun and phospho-JNK/cJun levels by Western blot. As demonstrated in Figure 3, phospho-JNK and phospho-c-Jun, as well as total c-Jun levels, were markedly increased in 4T1-RCKD cells compared to 4T1-pSMP control cells (Figure 3e, 3f, 3g, 3h). Surprisingly, JNK phosphorylation was also significantly increased in B16-RCKD cells. However, phospho-c-Jun and total c-Jun levels were significantly reduced in B16-RCKD clones compared to B16-pSMP clones (Figure 3e, 3f, 3g, 3h). In agreement with differential activation patterns of c-Jun in the two cell lines, the expression of cyclin D1, one of the c-Jun target genes, was also markedly reduced in B16-RCKD clone, but evidently increased in 4T1-RCKD cells, compared to their corresponding pSMP controls (Figure 3g). Collectively, our data demonstrate that IL-17RC silencing results in acquired JNK-activation in B16 and 4T1 cells but distinct c-Jun activities in the two tumor cell lines.

**Figure 4:**
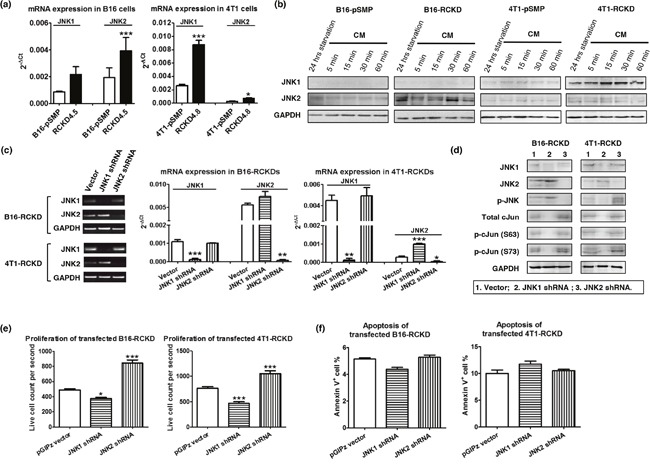
IL-17RC silencing induces acquired-activation of different JNK isoforms in different tumor cells, which differentially regulate c-Jun activities and homeostatic cell proliferation **(a)** JNK1 and JNK2 mRNA levels were determined by qRT-PCR in pSMP and RCKD subclones of B16 and 4T1 tumor cells. **(b)** Whole-cell extracts were harvested and immunoblotted to detect total JNK1 and JNK2 protein. GAPDH was used as a loading control. **(c-f)** RCKD subclones of B16 and 4T1 cells were transfected with shRNA targeting JNK1, JNK2 or vector alone. **(c)** JNK1 and JNK2 mRNA levels were determined by RT-PCR and qRT-PCR from day 3 transfected cells. **(d)** Day 3 transfected cells were serum-free starved for 14 hours and rescued with CM for 1 hour. Whole-cell extracts were harvested and immunoblotted to detect total or phosphorylated proteins. GAPDH was used as a loading control. **(e)** Day 3 transfected cells were seeded and cultured for an additional 3 days. Cells were then collected for flow cytometric analysis of their proliferation rate. For each sample, 20,000 events were collected and the time of collection was recorded. Propidium iodide (PI) was used to exclude the dead cells. The PI^−^ live cell count for each sample was divided by the respective time of collection to quantify the cell proliferation rate. **(f)** Quantified results of Annexin V^+^ cell percentage in B16 and 4T1 cultures. Values are presented as the mean ± SEM of 4-6 replicates in two independent experiments. *p ≤ 0.05; **p ≤ 0.01; ***p ≤ 0.001; statistical analysis was compared with the vector control.

Given that the IL-17RC silencing induced consistent JNK-activation with distinct c-Jun activities and inverse proliferation patterns in the two tumor models, we questioned whether IL-17RC silencing induced activation of different JNK isoforms in B16 and 4T1 cell lines. Indeed, IL-17RC silencing in B16 cells significantly increased mRNA and protein levels of JNK2 (Figure 4a, 4b). Conversely, IL-17RC silencing in 4T1 cells induced marked upregulation of JNK1 (Figure 4a, 4b). To further verify whether the distinct c-Jun activities and proliferation profiles observed in RCKD clones of B16 and 4T1 cells were due to differential expression/activation of JNK1 and JNK2 isoforms, we used another retroviral vector pGIPz to deliver shRNAs targeting endogenous *Jnk1* or *Jnk2* in B16-RCKD and 4T1-RCKD cells (Figure 4c, 4d). While both JNK1 and JNK2 shRNA displayed specific targeting effects in both B16-RCKD and 4T1-RCKD clones (Figure 4c, 4d), the JNK1 shRNA increased *Jnk2* mRNA and JNK2 protein in 4T1 cells (Figure 4c, 4d), indicating a potential role of JNK1 in repressing JNK2 expression under steady-state conditions. Importantly, the level of phospho-JNK in B16-RCKD and 4T1-RCKD clones was markedly attenuated by JNK2 and JNK1 shRNA, respectively (Figure 4d), reinforcing the notion that IL-17RC silencing induces differential acquired-activation of distinct JNK isoforms in the two tumor cell lines. Despite differential expression/activation of JNK isoforms in B16 and 4T1 cells, JNK1 shRNA was able to completely remove total and phospho-c-Jun signals, demonstrating a critical role of JNK1 in maintaining baseline c-Jun activities. Conversely, JNK2 shRNA enhanced total and phospho-c-Jun (S73) levels in B16-RCKD and, possibly, 4T1-RCKD cells, indicating a potential role of JNK2 in suppressing baseline c-Jun activities (Figure 4d). Importantly, JNK1 silencing consistently attenuated the proliferation of both B16-RCKD and 4T1-RCKD cells, whereas JNK2 silencing increased the proliferation of both cell lines (Figure 4e). Notably, the apoptosis rates were not significantly affected by JNK1/JNK2 silencing in both cell lines (Figure 4f). Taken together, our data suggest that IL-17RC silencing induces tumor-specific expression and activation of JNK1 and JNK2 isoforms, which have opposing roles in controlling downstream c-Jun activity and c-Jun-dependent homeostatic proliferation.

### IL-17RC is critically required for maintaining basal production of A20 that restrains homeostatic activation of JNK and NF-κB pathways

**Figure 5:**
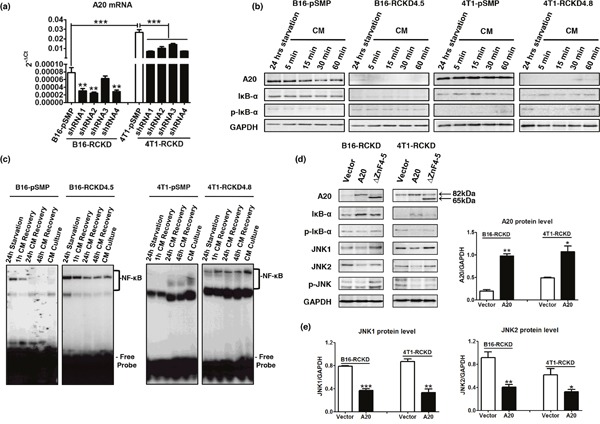
IL-17RC is critically required for maintaining basal production of A20 to repress homeostatic activities of JNK1 and JNK2 **(a)** A20 mRNA levels as determined by qRT-PCR in pSMP and RCKD subclones of B16 and 4T1 tumor cells. **(b)** Whole-cell extracts were harvested and immunoblotted to detect total or phosphorylated proteins, as indicated. GAPDH was used as a loading control. **(c)** Nuclear proteins (NPs) were extracted from RCKD and pSMP cells of B16 and 4T1 cells and subjected to EMSA using ^32^P-labeled NF-κB DNA probes. **(d)** RCKD cells were transfected with plasmid vector, plasmids expressing A20 or its mutant ΔZnF4-5 for 72 hours. Whole-cell extracts were harvested and immunoblotted to detect phosphorylated or total proteins as indicated. GAPDH was used as the loading control. **(e)** Scanning densitometry of relative protein levels was performed. Values are presented as the mean ± SEM of multiple replicates from two independent experiments. *p ≤ 0.05; **p ≤ 0.01; ***p ≤ 0.001; statistical analysis was compared with the respective vector control.

Having demonstrated a surprising role of IL-17RC in restraining activation of JNK in both B16 and 4T1 tumors, we questioned whether this observation was due to the loss of a critical negative control. Given the important role of A20 in negatively controlling multiple pathways, we examined A20 expression in RCKD and pSMP clones of B16 and 4T1 tumors. As demonstrated in Figure 5, A20 mRNA levels in RCKD clones of both B16 and 4T1 tumors were consistently reduced by four different IL-17RC-targeting shRNA constructs (Figure 5a). Notably, the basal level of A20 in B16-pSMP cells was significantly lower than that in 4T1-pSMP cells. In agreement with the mRNA profile, A20 protein levels were also consistently reduced in RCKD clones compared to pSMP cells under regular complete medium (CM) conditions or after 24 hours of serum-free starvation (Figure 5b), highlighting a critical role of IL-17RC in maintaining basal production of A20. In accordance with the role of A20 in negatively regulating the NF-κB pathway [26], NF-κB activity as measured by the phospho-IκB-α level and EMSA assays, demonstrated that the nuclear translocation of NF-κB was markedly and persistently elevated in RCKD clones compared to pSMP counterparts (Figure 5b, 5c).

To further verify that IL-17RC-controlled A20 was responsible for acquired-JNK activation in RCKD clones, we transfected RCKD clones with a plasmid carrying full length A20 or the empty plasmid vector and examined the intracellular signaling molecule profile by Western blot. While the A20 plasmid effectively restored A20 levels in RCKD clones, A20 reconstitution also successfully reduced the level of phopho-IκBα, total and phospho-JNK1 and JNK2 at 72 hours post-transfection in both B16-RCKD and 4T1-RCKD cells (Figure 5d, 5e), confirming that acquired homeostatic activation of both NF-κB and JNK pathways in RCKD clones was due to reduced A20 production (Figure 5d, 5e). Since the ZnF4-5 domain of A20 is critical for K48-mediated ASK1 degradation to inhibit TNF-induced JNK-c-Jun activation [32], we also used a ΔZnF4-5 mutant (~65kDa) plasmid to determine whether A20 may also utilize this mechanism in controlling homeostatic JNK activation. Notably, the ΔZnF4-5 mutant exhibited clear functional impairment in reducing JNK phosphorylation compared to WT A20 counterparts in both B16-RCKD and 4T1-RCKD clones, suggesting that A20 inhibits homeostatic JNK activation mainly through the ZnF4-5 domain. In comparison, the ΔZnF4-5 mutant exhibited less consistent functional alterations compared to WT A20 in controlling homeostatic NF-κB activity (Figure 5d).

### The IL-17RC-A20 axis is required to selectively repress cytokine production downstream of NF-κB and JNK-c-Jun pathways

**Figure 6:**
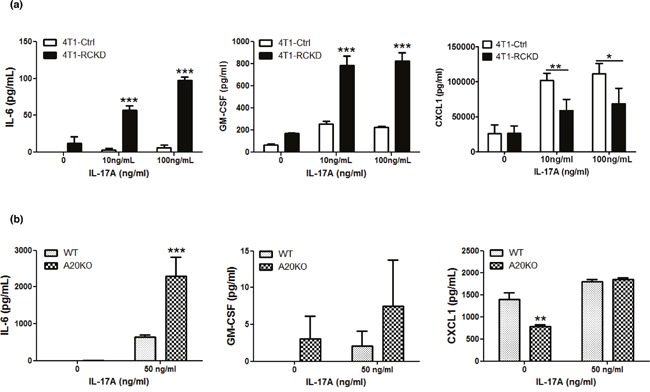
The IL-17RC-A20 axis is critically required to selectively repress cytokine production downstream of NF-kB and JNK-c-Jun pathways **(a)** IL-17RCKD and scramble control 4T1 cell lines were cultured or stimulated with recombinant IL-17A as indicated. Cytokine ELISAs were performed using day 3 culture supernatants. **(b)** WT and A20KO MEFs were stimulated with or without 50 ng/ml recombinant IL-17A. Cytokine ELISAs were performed using day 3 culture supernatants. Values are presented as the mean ± SEM of 3-5 independent experiments. *p ≤ 0.05; **p ≤ 0.01; ***p ≤ 0.001; statistical analysis was compared with the pSMP vector control in panel a, or, WT cells in panel b.

Having demonstrated that the basal levels of NF-κB and JNK pathways are up-regulated in RCKD cells, we hypothesized that, in addition to controlling homeostatic proliferation, the IL-17RC-A20 axis may also control secretion of pro-inflammatory cytokines downstream of NF-κB and JNK pathways under steady-state conditions and upon cytokine stimulation. To this end, we cultured 4T1-pSMP control and 4T1-RCKD cells in the absence or presence of recombinant IL-17A and measured the levels of GM-CSF and IL-6 as representative cytokines downstream of NF-κB and JNK pathways in the culture supernatants. Given the impaired CXCL1 production by RCKD cells upon IL-17A stimulation observed in our initial characterization experiments (Figure 1c), we also measured CXCL1 as a control. Notably, the levels of IL-6 and GM-CSF, but not CXCL1, showed a clear trend of enhanced basal production by 4T1-RCKD cells compared to the pSMP control cells. Despite very low levels of IL-17RC expression on the surface of RCKD cells, 4T1-RCKD cells actually produced significantly more IL-6 and GM-CSF, but not CXCL1, upon IL-17A stimulation (Figure 6a). A similar experiment was conducted using mouse embryonic fibroblasts (MEFs) isolated from C57BL/6 mice and A20-knockout (A20KO) mice. As demonstrated in Figure 6b, A20KO MEFs produced significantly more IL-6 and a higher trend of GM-CSF compared to WT counterparts. In comparison, a marked reduction of CXCL1 was observed in A20KO MEFs under steady-state condition while they produced similar amounts upon IL-17A stimulation. Collectively, our data indicates that, in addition to controlling tumor-specific proliferation, the IL-17RC-A20 axis has a regulatory role in selectively repressing production of pro-inflammatory cytokines, including IL-6 and GM-CSF, but not CXCL1.

## DISCUSSION

**Figure 7:**
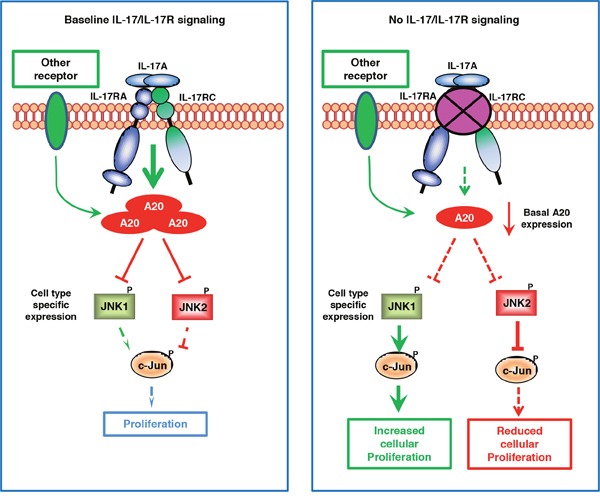
Proposed model of JNK1/JNK2 isoform-dependent tumor-specific proliferation controlled by baseline IL-17/IL-17R signaling Under steady-state conditions, IL-17R-dependent signaling and, to a lesser extent, IL-17R-independent signaling are required for maintaining baseline A20 production, which serves as a critical negative regulator for restraining the activation of both JNK1 and JNK2. When the baseline IL-17R signaling is severely diminished, the basal A20 production is markedly reduced, leading to aberrant activation of JNK1 or JNK2 in a cell type-dependent manner. When JNK1 is present as the dominant isoform, JNK1 induces c-Jun phosphorylation and promotes c-Jun-dependent proliferation; conversely, when JNK2 is present as the dominant isoform, JNK2 degrades c-Jun and suppresses c-Jun-dependent cell proliferation.

The role of the IL-17/IL-17R axis in cancer has been widely explored and conflicting results have been generated without a satisfactory explanation. By comparing IL-17RC silencing-induced functional and intracellular signaling changes in two different tumor cell lines, we have explicitly demonstrated a novel molecular mechanism underlying IL-17RC-controlled tumor-specific proliferation (see Figure 7 for our model). In contrast to the existing dogma that IL-17R-dependent signaling induces JNK activation, we provide solid evidence that IL-17RC is a key molecule in restraining homeostatic activation of JNK by maintaining baseline A20 production. Depending on the endogenous activities of JNK1 and JNK2, IL-17RC-dependent signaling may either positively or negatively regulate homeostatic proliferation and invasiveness of tumor cells.

Cancer-related chronic inflammation and pro-inflammatory cytokines play important roles in promoting aberrant activation of various signaling molecules, including NF-κB and JNK, in malignancies [43, 44]. In our study, we have unexpectedly discovered a novel scenario in which aberrant activation of NF-κB and JNKs in cancer cells is acquired upon the loss of a pro-inflammatory molecule—IL-17RC, and an anti-inflammatory molecule—A20. Therefore, for the first time, our study has united two distinct signals (pro-inflammatory and anti-inflammatory) into a single regulatory system and revealed an elegant “yin-yang” collaborative mechanism for controlling aberrant activation of transcription factors. Consistent with our finding, A20-deficient mice and mice with epidermis-specific A20-deficiency both display thickening of epidermal and dermal layers as the result of uncontrolled proliferation of keratinocytes [28, 45]. In the past, a two-phase model has been used to describe A20-mediated cytokine regulation, in which A20 expression is induced in the first phase by inflammatory stimuli like TNF-α, IL-1β, LPS and IL-17A, whereas A20 acts as a feedback inhibitor of its inducing inflammatory pathways in the second phase [46]. Our study has extended this model to the basal levels of IL-17RC-dependent signaling in controlling baseline A20 production and A20-dependent responses under steady-state conditions. Although the physiological relevance of our finding in human health and disease conditions remains to be determined, a recent study by Dr. Kolls's group has provided supporting evidence [47]. While conditional deletion of IL-17R in the mouse enteric epithelium caused reduced expression of α-defensins, *Pigr*, and *Nox1* which are required to control gut commensal bacteria, other anti-microbial peptides like family members of regenerating-islet derived (Reg) 3 family members (Reg3α/β/γ) were markedly increased in these mice, along with increased intestinal *Csf2* expression and elevated systemic GM-CSF cytokine production, as well as increased susceptibility to autoimmune responses [47]. Although an increased load of enteric segmented filamentous bacteria is likely to be an important trigger for over-expression of Reg3α/βγ and GM-CSF in the gut [47], we believe the loss of IL-17R-A20 regulation contributes to selective up-regulation of certain anti-microbial peptides and pro-inflammatory cytokine in this model.

Multiple intracellular molecules have been reported to directly or indirectly interact with IL-17RC under resting conditions. Gaffen's group has identified interactions of IL-17RC with anaphase-promoting complex protein 5 (APC5) and APC7 under steady-state conditions, which also directly interact with A20 [48]. Of note, the anaphase-promoting complex/cyclosome (APC/C) is a multi-subunit E3 ubiquitin ligase that targets more than 30 proteins for ubiquitin-dependent proteasome degradation and has an essential role in controlling the cell cycle [49]. APC5 and APC7 also interact directly with the transcription co-activators CBP/p300, stimulating intrinsic CBP/p300 acetyltransferase activity to potentiate transcription of gene targets, including A20 [50]. Notably, the general transcription apparatus and the CBP/p300 coactivators are indeed constitutively associated with the core promoter of A20 under basal conditions, which allows basal production of A20 [31]. Collectively, our results, in conjunction with other published studies, suggest that a basal level of A20 production may be maintained through a mechanism involving APC5/APC7 and CBP/p300. Of importance, once A20 levels are maintained, IL-17RC is unlikely to be required for A20 biological activities since reconstituted A20 was able to inhibit NF-κB and JNK activity in the absence of IL-17RC (Figure 5d). This may raise a question as to whether other pro-inflammatory signals are able to compensate for the IL-17RC-A20 axis and override IL-17RC silencing-induced A20 reduction and JNK-isoform-dependent cell proliferation *in vivo*. We believe this is not the case since IL-17RCKD clones of both B16 and 4T1 tumors show consistent invasiveness *in vitro* and *in vivo*. Nevertheless, this remains to be determined experimentally. Furthermore, it is intriguing whether other non-IL-17R inflammatory receptors including TNFR, IL-1R and TLR may also control tumor-specific proliferation in a manner that we have demonstrated for IL-17RC.

In addition to restraining homeostatic JNK activation, the basal cellular levels of A20 may also control IL-17A-induced JNK activation, given that IL-17A stimulation only induces JNK activation in selected cell types [18–21]. In our hands, although IL-17A was able to induce NF-κB activation and A20 production in both B16 and 4T1 cells, it only induced JNK activation in B16 but not 4T1 cells (Supplementary Figure 1). The selective IL-17A-induced activation of JNK in B16 cells is likely due to the lower basal levels of A20 in B16 cells compared to 4T1 cells, since 4T1 and B16 cells showed comparable JNK activation upon TGFβ stimulation (Supplementary Figure 1). Therefore, different from A20-mediated regulation of the NF-κB pathway, which only restricts the second phase of classic NF-κB activation as predicted by a mathematical model [51], A20 is able to control both the first and second phases of JNK activation. Most importantly, while our data indicates that both homeostatic activation of JNK and NF-κB pathways are restrained by the IL-17RC-A20 axis, it is the JNKs and not the NF-κB pathway that are responsible for controlling homeostatic tumor-dependent proliferation.

As a prototype of JNK-induced signaling pathway, the role of different JNK isoforms and the JNK-c-Jun axis in controlling cell cycle progression, cell proliferation and cell apoptosis has been extensively studied. Our data is highly consistent with the notion that JNK1 and JNK2 have opposing roles in controlling cell cycle progression and cell proliferation, with JNK1 and JNK2 being positive and negative regulators of these processes, respectively. Relevant to our study, specific gene knockdown of JNK1, but not JNK2, inhibits the growth of human melanoma cell lines [52]. JNK2 inhibits oncogene-induced breast cancer development *in vivo* by preventing cell cycle progression and DNA repair of breast cancer cells [53]. The opposing roles of JNK1 and JNK2 in regulating c-Jun dependent cell cycle progression was first observed in fibroblasts, erythroblasts and hepatocytes lacking JNK1 and/or JNK2 expression [38]. However, the notion of JNK2 being a negative regulator of c-Jun was challenged by the observation that JNK2 is fully able to phosphorylate c-Jun upon stimulation-induced activation [54]. Notably, in addition to activation of their substrates, the JNKs cause degradation of various substrate proteins, including c-Jun, ATF2, and p53, under non-stimulatory conditions [55]. The substrate degradation process is dependent on binding of the JNKs to the substrates and occurs in the absence of substrate phosphorylation [55]. Biochemically, JNK2 has a 25-fold higher binding affinity for c-Jun than JNK1, which is the major JNK isoform that binds to and constitutively degrades c-Jun under steady-state conditions [38, 56]. Upon stimulation, JNK1 becomes the major isoform to preferentially bind to c-Jun and induce c-Jun activation and c-Jun-dependent responses [38]. Therefore, the specific role of JNK1 and JNK2 in regulating c-Jun-dependent cell proliferation is context-dependent and stimulation-dependent. The opposing roles of JNK1 and JNK2 in controlling tumor cell proliferation are mediated through distinct molecular mechanisms and more applicable under steady-state conditions and, potentially, developing tumors during equilibrium phase.

In summary, our study elucidates a previously uncharacterized tumor-suppression/evasion mechanism whereby the basal levels of anti-inflammatory A20 is controlled by IL-17RC. Reduced IL-17RC expression can lead to aberrant JNK activation and altered tumor cell proliferation in a JNK-isoform-dependent manner. Effective cancer therapies may be devised by manipulating this novel control mechanism. Given that JNK1 and JNK2 perform antagonistic functions to mediate c-Jun-dependent basal proliferation of cancer cells, there is an urgent need to develop JNK isoform-specific inhibitors for cancer immunotherapies [35].

## MATERIALS AND METHODS

### Cell lines

Mouse B16 melanoma and 4T1 mammary carcinoma cells were originally purchased from ATCC (Manassas, VA). All tumor cell lines were maintained in complete DMEM medium. Phoenix cells were obtained from Dr. Craig McCormick (Dalhousie University) and cultured in complete MEM F11 medium.

### Retroviral vectors and DNA plasmids

Recombinant retroviruses that encode shRNA sequences targeting four different regions of IL-17RC or scrambled gene sequences were constructed using pSMP vector (Open Biosystems). The shRNA constructs were mixed with polyethyleneimine 40,000 MW (Polysciences Inc.) and used to transfect Phoenix cells for 3 days. The virus-containing culture supernatants were used to transduce B16 and 4T1 tumor cells. Stable transfectants were selected by treating cells with 4 μg/ml puromycin (Bio Basic Inc.) for 7 days or until all non-transfected tumor cells died. Selected cells were subjected to a limiting-dilution-assay to obtain single-cell-derived subclones. Ten subclones of each shRNA construct were expanded for further analyses.

In some experiments, GIPZ lentiviral shRNAs (Thermo Scientific), including JNK1 (V2LMM_49133) and JNK2 (V3LMM_472591, V3LMM_515242 and V3LMM_515241), were used to knockdown JNK1 or JNK2 in IL-17RCKD tumor cells. At day 3 post-transfection, tumor cells were starved in serum-free medium for 14 hours and then rescued with complete medium (CM) for 1 hour. Whole-cell extracts were harvested and the level of JNK1 or JNK2 protein was examined using Western blotting.

In some experiments, A20 reconstitution was conducted using plasmids encoding murine A20 or a deletion-mutant, which were purchased from the plasmid repository at BCCM/LMBP (Belgian Coordinated Collections of Micro-organisms and Laboratory of Molecular Biology–Plasmid collection).

### RNA extraction, RT-PCR, and quantitative real-time PCR (qRT-PCR)

Total RNAs were extracted from 3 × 10^6^ tumor cells using RNeasy columns (Qiagen) and first strand cDNA was generated using a QuantiTect Reverse Transcription kit (Qiagen). PCR reactions were performed with gene-specific primers (Supplementary Table 1) using PCR Master Mix (Promega) in an Eppendorf Mastercycler PCR machine. For qRT-PCR, cDNA was amplified using RT^2^ SYBR® Green ROX qPCR Mastermix (Qiagen) in a 7900 HT Fast Real-Time PCR System (Applied Biosystems, Foster City, USA). Each amplification contained no-cDNA control wells and positive control wells using XpressRef™ Mouse Universal Total RNA (Qiagen). *GAPDH* was used as an internal normalization control. The data were analyzed using the SDS software 2.2.2 from Applied Biosystems.

### MTT proliferation assay

Tumor cells were plated in quadruplicate in a 96-well plate at a density of 5,000 cells per well. Cells were incubated at 37°C for the indicated times. At the end of assay, cells were incubated with 0.5 mg/ml MTT for 2 hours and the purple formazan products were dissolved in 100 μL of DMSO. The plates were read on a plate reader at 570 nm with a reference reading at 630 nm. OD values collected at 6 hour post seeding were used for normalizing the proliferation rate at different time points. In some assays, the cells were treated with different chemical inhibitors (Supplementary Table 2) or the DMSO vehicle. The inhibitors SB203580, FR180204, 420116 and SP600125 were purchased from EMD Millipore. KIN001-102 and BMS-345541 were purchased from Sigma. All inhibitors were reconstituted in dimethyl sulfoxide (DMSO).

### *In vivo* tumor model

C57BL/6 male and BALB/c female mice (8-10 weeks) were purchased from Charles River Laboratories (Senneville, QC). Mice were housed in the IWK Health Centre animal facility under pathogen-free conditions. All animal procedures were approved by the University Committee on Laboratory Animal of Dalhousie University.

For the B16 melanoma model, C57BL/6 mice received 1×10^6^ of B16-RCKD or control tumor cells in 100 μl of serum-free DMEM medium by subcutaneous injection in the hind leg. Mice were sacrificed at day 12 and the tumors were collected and weighted. For the 4T1 mammary carcinoma model, BALB/c mice were inoculated with 1×10^6^ of 4T1-RCKD or control tumor cells in 100 μl of serum-free DMEM medium in the left side of fourth mammary fat pad. At day 18 post-inoculation, mice were sacrificed and lung metastases were quantified using a colony-forming assay. Tumor growth in both animal models was monitored every 4 days by engineer's caliper and the volume was calculated as V= (W^2^ × L)/2.

### Quantification of lung metastases by colony-forming assay

Lungs were aseptically removed from mice and placed in HBSS. After mincing with scissors, lungs were digested in 5 ml of HBSS containing 1 mg/ml collagenase IV (Sigma-Aldrich, St Louis, MO), to which 10 units of elastase (Sigma-Aldrich) were added directly. The lungs were digested for 75 minutes at 4°C on a rotating wheel and then filtered through 70 μm cell strainers, and washed twice with RPMI 1640 medium containing 10% FBS. Single-cell suspensions were re-suspended in complete RPMI 1640 medium supplemented with 60 μM 6-thioguanine, and seeded into 100 mm tissue culture plates. Cells were incubated at 37°C for 10-14 days until tumor colonies were visible. The colonies were then fixed with 100% methanol for five minutes and washed with distilled water. The fixed colonies were stained with 5 ml of 0.03% methylene blue stain and counted by naked eye. Data are expressed as total number of metastatic colonies per lung.

### Immunohistochemistry staining and ImageJ densitometry

For immunostaining, 5 μm-sections of tumors were deparaffinized and incubated with the primary antibody recognizing cleaved caspase-3 (Cell Signaling no. 9664) overnight at 4°C. The slides were washed and then incubated with biotinylated goat anti-rabbit IgG and followed by a peroxidase conjugate ABC solution (Vector Laboratories). The slides were developed by the addition of diaminobenzidine in DAB solution (Vector Laboratories) and counterstained with Mayer's Haematoxylin. The slides were then immersed in Scott's solution and mounted with a coverslip. After overnight drying, images of each sample were captured using LEICA Application Suite (version 2.5.0 R1) with an optimized condition. The percent area of positive staining for each image was recorded and calculated by densitometry analysis using ImageJ.

### Western blot

Western blotting was conducted using primary antibodies recognizing JNK1 (Santa Cruz Biotechnology no. sc-1648), JNK2 (Santa Cruz Biotechnology no. sc-827), total JNK (Cell Signaling no. 9252), phospho-JNK (Thr183/185; Cell Signaling no. 4668), total c-Jun (Cell Signaling no. 9165), phospho-c-Jun (Ser63; Cell Signaling no. 2361), phospho-c-Jun (Ser73; Cell Signaling no. 3270), A20/TNFAIP3 (Cell Signaling no. 5630), IκB-α (Cell Signaling no. 4814), phospho-IκB-α (Cell Signaling no. 9246), caspase-3 (Cell Signaling no. 9662), cleaved caspase-3 (Cell Signaling no. 9664) or GAPDH (Cell Signaling no. 5174). All antibodies were diluted in PBS-Tween (0.1%). The intensities of bands of interest were analyzed using ImageJ software.

### EMSA

Nuclear protein extracts were prepared using a nuclear extract kit (Active Motif). EMSA was performed using a ^32^P-labled oligonucleotide probe specific for the NF-κB consensus sequence on the IL-6 promoter [57]. Briefly, 10 μg of nuclear protein was added to 10 μl of binding buffer supplemented with 1 μg poly-(dI-dC) (GE Healthcare) and incubated at room temperature for 15 minutes before mixing with the probe. The reaction mixture was incubated at room temperature for 30 minutes and subjected to electrophoresis on 6% polyacrylamide gels. Gels were vacuum-dried and subjected to autoradiography.

### Apoptosis assay

Tumor cell apoptosis was assessed using the Annexin V-FITC Apoptosis Detection Kit (eBioscience). Data were acquired on a Becton Dickinson FACSCalibur and analyzed using FCS Express 4 (De Novo).

### Cytokines ELISA

CXCL1, IL-6 and GM-CSF were measured by ELISA kits purchased from eBioscience. The optical density (OD) was read at 450 nm using a BioTek synergy reader (BioTek).

### Statistical analysis

Data were expressed as means ± the standard error of the mean. Statistical analyses were done using GraphPad Prism version 5.0 (GraphPad Software). Correlations between groups were analyzed by Pearson's and Spearman's correlation coefficient. The two-tailed unpaired student *t* test was used to determine the significance of the differences between groups. For comparison of multiple groups, the ANOVA test followed by multiple comparisons of means was applied. The *p* values ≤ 0.05 were considered statistically significant.

## SUPPLEMENTARY FIGURE AND TABLES



## Abbreviations

APC5: anaphase promoting complex protein 5
APC7: anaphase promoting complex protein 7
APC/C: anaphase promoting complex/cyclosome
Akt: serine/threonine-specific protein kinase, also known as protein kinase B
CM: complete medium
ERK: extracellular signal-regulated kinase
GM-CSF: granulocyte- macrophage colony-stimulating factor
IKK: Ikappa B kinase
JNKs: c-Jun NH2-terminal kinases
KD: knockdown
KO: knockout
MAPK: mitogen-activated protein kinase
MEFs: mouse embryonic fibroblasts
MTT: 3-(4,5-Dimethylthiazol-2-yl)-2,5-diphenyltetrazolium bromide for
NF-κB: nuclear factor kappa B
Reg3: regenerating-islet derived 3
SF: serum-free
TNFAIP3: TNFα-induced protein 3
PI3K: phosphatidylinositide 3-kinases
