# Supplementary material for: IL-17RC is critically required to maintain baseline A20 production to repress JNK isoform-dependent tumor-specific proliferation
Source: Oncotarget. 2017 May 11;8(26):43153–68. doi: 10.18632/oncotarget.17820 (PMC5522135; doi:10.18632/oncotarget.17820)
Supplement: Supplementary file 1 [file oncotarget-08-43153-s001.pdf]

# IL-17RC is critically required to maintain baseline A20 production to repress JNK isoform-dependent tumor-specific proliferation

## SUPPLEMENTARY MATERIALS

## SUPPLEMENTARY FIGURE AND TABLES

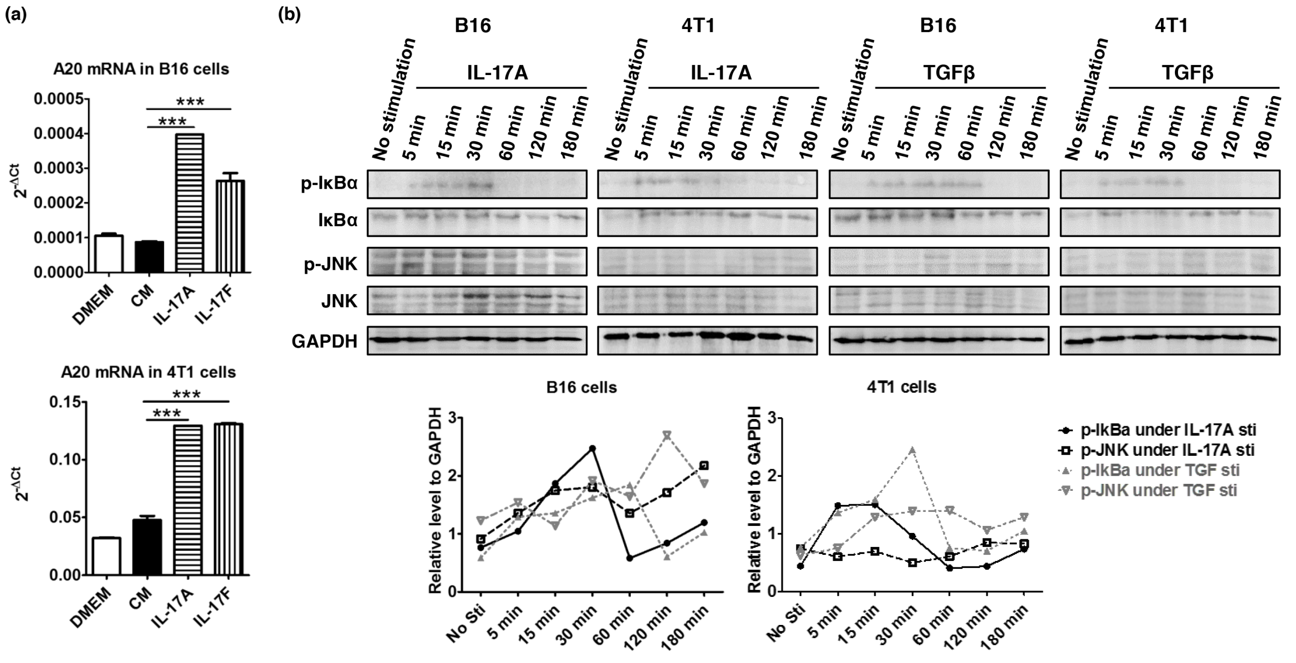


**Supplementary Figure 1: IL-17A triggers A20 and NF-κB induction and cell-type-dependent JNK activation. (a)** B16 melanoma and 4T1 breast cancer cells were starved with serum-free DMEM medium for 14hrs and recovered with complete DMEM medium (CM) with or without exogenous 200ng/ml IL-17A or IL-17F for 30mins. Gene expression was determined by qRT-PCR. Values were presented as the mean ± SEM of 6 replicates from two independent experiments. ***$p \leq 0.001$; statistical analysis was compared with the CM group. **(b)** Tumor cells were treated with or without 50ng/ml IL-17A or 5ng/ml TGF-β for the time indicated. Whole-cell extracts were harvested and immunoblotted to detect total or phosphorylated proteins as indicated. GAPDH was used as a loading control.

**Supplementary Table 1: Primers used in this study**

| Gene* | Sequence (5'→3') | TM (°C) | Length |
|---|---|---|---|
| mIL-17RA sense | AGGGCTGCGGCATGTGAT | 60.4 | 140bp |
| mIL-17RA antisense | GCCTCCCGAGTTCTCCTGTTA | 58.5 | |
| mIL-17RC sense | AGATGCCTGTGTCCTGGTTC | 57.1 | 243bp |
| mIL-17RC antisense | CGCAATCTGTCTTCTGTGGA | 54.9 | |
| mA20 sense | AATCGGCTGCTTCCTATGACTC | 60.3 | 236bp |
| mA20 antisense | CTTCCTCGTCCTCACGGCTA | 60.2 | |
| mJNK1 sense | GGAGGTAATGGATTTGGAGGA | 58.1 | 119bp |
| mJNK1 antisense | ACAGACGGCGAAGACGATG | 59.0 | |
| mJNK2 sense | ACACGAATAGATGTTGAAGTGTCG | 58.9 | 203bp |
| mJNK2 antisense | TTGGCAGGTTCTCCTGGTTA | 58.0 | |
| mGAPDH sense | CGATGCCCCCATGTTTGTGAT | 58.2 | 249bp |
| mGAPDH antisense | GCAGGGATGATGTTCTG | 55.8 | |

**Supplementary Table 2: Inhibitors used in this study and their properties**

| Inhibitor | Target pathway | Mode of action |
|---|---|---|
| KIN001-102 | AKT | Isozyme selective Akt1/2 kinase inhibitor |
| BMS-345541 | NF-κB | Highly selective I kappa B kinase (IKK) allosteric site inhibitor |
| SB203580 | p38 MAPK | Competitive inhibition of ATP binding site of p38 MAPK |
| FR180204 | ERK 1/2 MAPK | Noncompetitive inhibition of IKKα and MEK 1/2 with respect to ATP/ERK |
| 420116 | JNK MAPK | Noncompetitive inhibition of ERK/P38 with inhibition to JNK phosphorylation |
| SP600125 | JNK/c-Jun MAPK | Competitive inhibition of JNK and c-Jun phosphorylation |

Note: The inhibitors SB203580, FR180204, 420116 and SP600125 were purchased from EMD Millipore. KIN001-102 and BMS-345541 were purchased from Sigma.
